# 4-Ethyl-1-(4-meth­oxy­benzyl­idene)thio­semicarbazide

**DOI:** 10.1107/S1600536810022919

**Published:** 2010-06-18

**Authors:** Yu-Feng Li, Fang-Fang Jian

**Affiliations:** a Microscale Science Institute, Department of Chemistry and Chemical Engineering, Weifang University, Weifang 261061, People’s Republic of China; b Microscale Science Institute, Weifang University, Weifang 261061, People’s Republic of China

## Abstract

In the title compound, C_11_H_15_N_3_OS, the dihedral angle between the aromatic ring and the thio­urea unit is 4.28 (7)° and an intra­molecular N—H⋯N hydrogen bond generates an *S*(5) ring. In the crystal, mol­ecules are linked into (001) sheets by N—H⋯S hydrogen bonds.

## Related literature

For background to the reactions and properties of thio­semicarbazones, see: Casas *et al.* (2000 ▶); Lobana *et al.* (2009 ▶); Quiroga & Ranninger (2004 ▶). For a related structure, see: Li & Jian (2010 ▶).
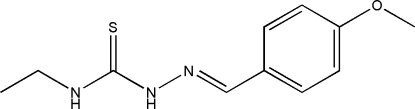


## Experimental

### 

#### Crystal data

C_11_H_15_N_3_OS
*M* *_r_* = 237.32
Orthorhombic, 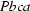

*a* = 13.066 (3) Å
*b* = 10.128 (2) Å
*c* = 19.224 (4) Å
*V* = 2543.9 (9) Å^3^
*Z* = 8
Mo *K*α radiation
μ = 0.24 mm^−1^
*T* = 293 K
0.22 × 0.20 × 0.18 mm

#### Data collection

Bruker SMART CCD diffractometer
22633 measured reflections
2912 independent reflections
2302 reflections with *I* > 2σ(*I*)
*R*_int_ = 0.044

#### Refinement

*R*[*F* ^2^ > 2σ(*F* ^2^)] = 0.053
*wR*(*F* ^2^) = 0.160
*S* = 1.05
2912 reflections
145 parameters
H-atom parameters constrained
Δρ_max_ = 0.26 e Å^−3^
Δρ_min_ = −0.34 e Å^−3^

### 

Data collection: *SMART* (Bruker, 1997 ▶); cell refinement: *SAINT* (Bruker, 1997 ▶); data reduction: *SAINT*; program(s) used to solve structure: *SHELXS97* (Sheldrick, 2008 ▶); program(s) used to refine structure: *SHELXL97* (Sheldrick, 2008 ▶); molecular graphics: *SHELXTL* (Sheldrick, 2008 ▶); software used to prepare material for publication: *SHELXTL*.

## Supplementary Material

Crystal structure: contains datablocks global, I. DOI: 10.1107/S1600536810022919/hb5492sup1.cif

Structure factors: contains datablocks I. DOI: 10.1107/S1600536810022919/hb5492Isup2.hkl

Additional supplementary materials:  crystallographic information; 3D view; checkCIF report

## Figures and Tables

**Table 1:** Hydrogen-bond geometry (Å, °)

*D*—H⋯*A*	*D*—H	H⋯*A*	*D*⋯*A*	*D*—H⋯*A*
N1—H1*A*⋯S1^i^	0.86	2.60	3.4080 (17)	156
N3—H3*A*⋯N2	0.86	2.26	2.634 (2)	106
N3—H3*A*⋯S1^ii^	0.86	2.78	3.4670 (17)	137

Symmetry codes: (i) 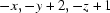; (ii) 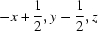.

**Fig. 1.**
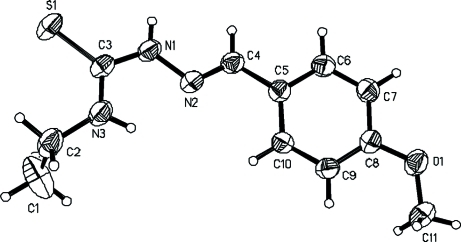
The structure of (I) showing 30% probability displacement ellipsoids.

## supplementary crystallographic information

### Comment

Thiosemicarbazones have attracted much attention because they can be utilized as effective ligands to form the compounds with antitumoral drugs. (Quiroga & Ranninger, 2004).They are important versatile coordination agents which have been reported to be coordination compounds (Casas *et al.*, 2000) (Lobana *et al.*, 2009). As part of our search for new thiosemicarbazones compounds we synthesized the title compound (I), and describe its structure here. The dihedral angle between the benzene ring and the thiourea unit is [4.28 (7)°]. Intermolecular N—H···S hydrogen bonds generate chains.

Bond lengths and angles agree with those observed in a related compound (Li & Jian, 2010).

### Experimental

A mixture of 4-methoxybenzaldehyde (0.1 mol) and 4-ethylthiosemicarbazide (0.1 mol) was stirred in refluxing ethanol (20 ml) for 2 h to afford the title compound (0.086 mol, yield 86%).  Colourless blocks of (I) were obtained by recrystallization from ethanol at room temperature.

### Refinement

H atoms were fixed geometrically and allowed to ride on their attached atoms, with C—H = 0.97 Å, and with *U*_iso_(H) = 1.2–1.5*U*_eq_(C).

### Crystal data

**Table:**

C_11_H_15_N_3_OS	*F*(000) = 1008
*M**_r_* = 237.32	*D*_x_ = 1.239 Mg m^−^^3^
Orthorhombic, *P**b**c**a*	Mo *K*α radiation, λ = 0.71073 Å
Hall symbol: -P 2ac 2ab	Cell parameters from 2302 reflections
*a* = 13.066 (3) Å	θ = 2.8–25.3°
*b* = 10.128 (2) Å	µ = 0.24 mm^−^^1^
*c* = 19.224 (4) Å	*T* = 293 K
*V* = 2543.9 (9) Å^3^	Block, colorless
*Z* = 8	0.22 × 0.20 × 0.18 mm

### Data collection

**Table:**

Bruker SMART CCD diffractometer	2302 reflections with *I* > 2σ(*I*)
Radiation source: fine-focus sealed tube	*R*_int_ = 0.044
graphite	θ_max_ = 27.5°, θ_min_ = 3.1°
phi and ω scans	*h* = −16→16
22633 measured reflections	*k* = −13→13
2912 independent reflections	*l* = −24→24

### Refinement

**Table:**

Refinement on *F*^2^	Primary atom site location: structure-invariant direct methods
Least-squares matrix: full	Secondary atom site location: difference Fourier map
*R*[*F*^2^ > 2σ(*F*^2^)] = 0.053	Hydrogen site location: inferred from neighbouring sites
*wR*(*F*^2^) = 0.160	H-atom parameters constrained
*S* = 1.05	*w* = 1/[σ^2^(*F*_o_^2^) + (0.0997*P*)^2^ + 0.2868*P*] where *P* = (*F*_o_^2^ + 2*F*_c_^2^)/3
2912 reflections	(Δ/σ)_max_ = 0.001
145 parameters	Δρ_max_ = 0.26 e Å^−^^3^
0 restraints	Δρ_min_ = −0.34 e Å^−^^3^

### Special details

**Table:**

Geometry. All e.s.d.'s (except the e.s.d. in the dihedral angle between two l.s. planes) are estimated using the full covariance matrix. The cell e.s.d.'s are taken into account individually in the estimation of e.s.d.'s in distances, angles and torsion angles; correlations between e.s.d.'s in cell parameters are only used when they are defined by crystal symmetry. An approximate (isotropic) treatment of cell e.s.d.'s is used for estimating e.s.d.'s involving l.s. planes.
Refinement. Refinement of *F*^2^ against ALL reflections. The weighted *R*-factor *wR* and goodness of fit *S* are based on *F*^2^, conventional *R*-factors *R* are based on *F*, with *F* set to zero for negative *F*^2^. The threshold expression of *F*^2^ > σ(*F*^2^) is used only for calculating *R*-factors(gt) *etc*. and is not relevant to the choice of reflections for refinement. *R*-factors based on *F*^2^ are statistically about twice as large as those based on *F*, and *R*- factors based on ALL data will be even larger.

### Fractional atomic coordinates and isotropic or equivalent isotropic displacement parameters (Å^2^)

**Table:**

	*x*	*y*	*z*	*U*_iso_*/*U*_eq_	
S1	0.12047 (4)	1.04218 (4)	0.57631 (3)	0.0768 (2)	
N1	0.09565 (11)	0.83299 (14)	0.49802 (8)	0.0648 (4)	
H1A	0.0548	0.8818	0.4742	0.078*	
N2	0.11117 (10)	0.70368 (14)	0.47913 (8)	0.0598 (3)	
C9	0.12596 (13)	0.30355 (18)	0.41061 (9)	0.0637 (4)	
H9A	0.1545	0.2367	0.4375	0.076*	
C3	0.14420 (12)	0.88334 (15)	0.55374 (9)	0.0596 (4)	
N3	0.21067 (12)	0.80566 (14)	0.58550 (9)	0.0719 (4)	
H3A	0.2271	0.7331	0.5651	0.086*	
O1	0.09603 (11)	0.15651 (14)	0.31258 (7)	0.0803 (4)	
C5	0.07418 (12)	0.53144 (16)	0.39807 (8)	0.0576 (4)	
C10	0.11699 (13)	0.42999 (18)	0.43678 (9)	0.0621 (4)	
H10A	0.1403	0.4476	0.4815	0.075*	
C4	0.06596 (13)	0.66625 (17)	0.42425 (9)	0.0626 (4)	
H4A	0.0262	0.7267	0.3999	0.075*	
C8	0.09189 (12)	0.27751 (17)	0.34373 (9)	0.0621 (4)	
C6	0.04087 (15)	0.5032 (2)	0.33114 (10)	0.0719 (5)	
H6A	0.0115	0.5696	0.3044	0.086*	
C11	0.14227 (19)	0.05082 (19)	0.34942 (13)	0.0866 (6)	
H11A	0.1397	−0.0278	0.3216	0.130*	
H11B	0.1061	0.0363	0.3922	0.130*	
H11C	0.2123	0.0725	0.3594	0.130*	
C2	0.25846 (16)	0.8338 (2)	0.65277 (13)	0.0910 (7)	
H2B	0.2628	0.9285	0.6594	0.109*	
H2C	0.3275	0.7984	0.6532	0.109*	
C7	0.05071 (16)	0.3784 (2)	0.30398 (10)	0.0769 (5)	
H7A	0.0296	0.3616	0.2586	0.092*	
C1	0.1984 (3)	0.7743 (4)	0.71090 (15)	0.1455 (13)	
H1B	0.2311	0.7943	0.7544	0.218*	
H1C	0.1952	0.6803	0.7049	0.218*	
H1D	0.1304	0.8100	0.7108	0.218*	

### Atomic displacement parameters (Å^2^)

**Table:**

	*U*^11^	*U*^22^	*U*^33^	*U*^12^	*U*^13^	*U*^23^
S1	0.0653 (3)	0.0441 (3)	0.1211 (5)	0.00297 (16)	−0.0100 (2)	−0.0109 (2)
N1	0.0687 (8)	0.0469 (7)	0.0786 (9)	0.0078 (6)	−0.0085 (7)	0.0005 (6)
N2	0.0596 (7)	0.0479 (7)	0.0720 (8)	0.0022 (5)	−0.0015 (6)	−0.0004 (6)
C9	0.0672 (9)	0.0567 (10)	0.0673 (9)	0.0013 (7)	−0.0052 (7)	0.0071 (7)
C3	0.0526 (7)	0.0447 (8)	0.0814 (10)	−0.0024 (6)	0.0014 (7)	−0.0002 (7)
N3	0.0716 (9)	0.0512 (8)	0.0929 (10)	0.0102 (6)	−0.0169 (8)	−0.0137 (7)
O1	0.0857 (8)	0.0647 (8)	0.0905 (9)	0.0036 (6)	−0.0126 (7)	−0.0171 (7)
C5	0.0515 (8)	0.0583 (9)	0.0632 (8)	0.0022 (6)	−0.0032 (6)	−0.0007 (6)
C10	0.0695 (10)	0.0594 (10)	0.0575 (8)	−0.0005 (7)	−0.0070 (7)	0.0017 (7)
C4	0.0604 (9)	0.0585 (9)	0.0688 (9)	0.0067 (7)	−0.0063 (7)	0.0016 (7)
C8	0.0547 (8)	0.0613 (10)	0.0702 (9)	−0.0008 (7)	−0.0022 (7)	−0.0071 (7)
C6	0.0762 (11)	0.0698 (10)	0.0697 (10)	0.0143 (9)	−0.0188 (8)	0.0012 (8)
C11	0.0946 (14)	0.0568 (11)	0.1083 (16)	0.0024 (9)	0.0065 (13)	−0.0021 (10)
C2	0.0766 (12)	0.0677 (12)	0.1285 (18)	0.0107 (9)	−0.0402 (13)	−0.0265 (11)
C7	0.0856 (12)	0.0772 (12)	0.0679 (10)	0.0109 (9)	−0.0231 (9)	−0.0102 (9)
C1	0.120 (2)	0.232 (4)	0.0849 (16)	−0.025 (2)	−0.0161 (16)	−0.032 (2)

### Geometric parameters (Å, °)

**Table:**

S1—C3	1.6948 (17)	C10—H10A	0.9300
N1—C3	1.345 (2)	C4—H4A	0.9300
N1—N2	1.3742 (19)	C8—C7	1.385 (3)
N1—H1A	0.8600	C6—C7	1.373 (3)
N2—C4	1.267 (2)	C6—H6A	0.9300
C9—C10	1.381 (2)	C11—H11A	0.9600
C9—C8	1.386 (3)	C11—H11B	0.9600
C9—H9A	0.9300	C11—H11C	0.9600
C3—N3	1.321 (2)	C2—C1	1.492 (4)
N3—C2	1.464 (2)	C2—H2B	0.9700
N3—H3A	0.8600	C2—H2C	0.9700
O1—C8	1.365 (2)	C7—H7A	0.9300
O1—C11	1.419 (3)	C1—H1B	0.9600
C5—C10	1.387 (2)	C1—H1C	0.9600
C5—C6	1.388 (2)	C1—H1D	0.9600
C5—C4	1.459 (2)		
			
C3—N1—N2	120.14 (13)	C7—C8—C9	119.75 (16)
C3—N1—H1A	119.9	C7—C6—C5	120.86 (16)
N2—N1—H1A	119.9	C7—C6—H6A	119.6
C4—N2—N1	115.87 (14)	C5—C6—H6A	119.6
C10—C9—C8	119.16 (16)	O1—C11—H11A	109.5
C10—C9—H9A	120.4	O1—C11—H11B	109.5
C8—C9—H9A	120.4	H11A—C11—H11B	109.5
N3—C3—N1	116.89 (14)	O1—C11—H11C	109.5
N3—C3—S1	124.54 (13)	H11A—C11—H11C	109.5
N1—C3—S1	118.51 (12)	H11B—C11—H11C	109.5
C3—N3—C2	124.95 (15)	N3—C2—C1	111.03 (19)
C3—N3—H3A	117.5	N3—C2—H2B	109.4
C2—N3—H3A	117.5	C1—C2—H2B	109.4
C8—O1—C11	118.37 (16)	N3—C2—H2C	109.4
C10—C5—C6	118.11 (16)	C1—C2—H2C	109.4
C10—C5—C4	122.54 (15)	H2B—C2—H2C	108.0
C6—C5—C4	119.32 (15)	C6—C7—C8	120.37 (16)
C9—C10—C5	121.73 (16)	C6—C7—H7A	119.8
C9—C10—H10A	119.1	C8—C7—H7A	119.8
C5—C10—H10A	119.1	C2—C1—H1B	109.5
N2—C4—C5	122.20 (15)	C2—C1—H1C	109.5
N2—C4—H4A	118.9	H1B—C1—H1C	109.5
C5—C4—H4A	118.9	C2—C1—H1D	109.5
O1—C8—C7	115.83 (15)	H1B—C1—H1D	109.5
O1—C8—C9	124.41 (16)	H1C—C1—H1D	109.5

### Hydrogen-bond geometry (Å, °)

**Table:**

*D*—H···*A*	*D*—H	H···*A*	*D*···*A*	*D*—H···*A*
N1—H1A···S1^i^	0.86	2.60	3.4080 (17)	156
N3—H3A···N2	0.86	2.26	2.634 (2)	106
N3—H3A···S1^ii^	0.86	2.78	3.4670 (17)	137

Symmetry codes: (i) −*x*, −*y*+2, −*z*+1; (ii) −*x*+1/2, *y*−1/2, *z*.

## References

[bb1] Bruker (1997). *SMART* and *SAINT* Bruker AXS Inc., Madison, Wisconsin, USA.

[bb2] Casas, J. S., Garcia-T, M. S. & Sordo, J. (2000). *Coord. Chem. Rev.* **209**, 197–261.

[bb3] Li, Y.-F. & Jian, F.-F. (2010). *Acta Cryst.* E**66**, o1399. 10.1107/S1600536810017988 PMC2979434 21579478

[bb4] Lobana, T. S., Khanna, S., Hundal, G., Kaur, P., Thakur, B., Attri, S. & Butcher, R. J. (2009). *Polyhedron*, **28**, 1583–1593.

[bb5] Quiroga, A. G. & Ranninger, C. N. (2004). *Coord. Chem. Rev.* **248**, 119–133.

[bb6] Sheldrick, G. M. (2008). *Acta Cryst.* A**64**, 112–122. 10.1107/S0108767307043930 18156677

